# Particularities of Cataract Surgery in Elderly Patients: Corneal Structure and Endothelial Morphological Changes after Phacoemulsification

**DOI:** 10.3390/geriatrics9030077

**Published:** 2024-06-08

**Authors:** Adela Laura Ciorba, Alin Teusdea, George Roiu, Daniela Simona Cavalu

**Affiliations:** 1 Doctoral School of Biomedical Sciences, Faculty of Medicine and Pharmacy, University of Oradea, P-ta 1 Decembrie 10, 410087 Oradea, Romania; adela.ciorba@yahoo.com (A.L.C.); daniela.cavalu@didactic.uoradea.ro (D.S.C.); 2 Faculty of Environmental Protection, University of Oradea, 26 Gen. Magheru Street, 410048 Oradea, Romania; 3 Faculty of Medicine and Pharmacy, University of Oradea, P-ta 1 Decembrie 10, 410087 Oradea, Romania

**Keywords:** phacoemulsification, specular microscopy, cell density, corneal endothelial morphology, central corneal thickness, cataract

## Abstract

The aim of this study was to evaluate the influence of ultrasounds used in phacoemulsification during cataract surgery on the corneal structure and morphology in patients over 65 years. We compared the outcomes of phacoemulsification techniques in terms of corneal cell morphology in 77 patients over 65 years old and 43 patients under 65 years old. Corneal cell density, central corneal thickness and hexagonality were measured preoperatively and post-surgery (at 1 and 4 weeks) by specular microscopy. The effect of gender, axial length and anterior chamber depth on the parameters of corneal endothelium were evaluated. In both groups, a progressive decrease in endothelial cells was observed, starting from the first week post-surgery until the fourth postoperative week. The central corneal thickness increased in both groups with maximum values at the first week postoperatively, while their initial values were restored in the fourth week post-surgery, with no statistical difference between groups. Statistically significant differences were noticed in terms of cell hexagonality in the group over 65, showing smaller hexagonality at all preoperative and postoperative time points compared to group under 65. Our result highlights the importance of routine specular microscopy performed before surgery, regardless the age of the patients, with caution and careful attention to the phaco power intensity, ultrasound energy consumption and intraoperative manipulation of instruments, as well as proper use of viscoelastic substances to reduce corneal endothelium damage, especially in elderly patients.

## 1. Introduction

According to a recent meta-analysis of population-based surveys of eye disease, the leading cause of blindness in people over 50 is cataract, followed by glaucoma, uncorrected refractive error, aged-related macular degeneration and diabetic retinopathy [1]. Cataract is considered a multifactorial eye disease, due to the opacification of the lens, that leads to visual impairment when is located in the visual axis. The main cause for cataract development is ageing together with oxidative stress. However, ageing is not a homogenous process and so the crystalline lens responds asymmetrically to its effects generating different degrees of lens opacification in patients of the same age category [2]. In developing countries, the incidence of cataract is high among the young generation, mainly due to malnutrition, but environmental and genetic factors are also explanatory factors [3]. On the other hand, in developed countries, age-related cataract affecting the population over 65 years old is a major concern [4]. Risk factors associated with cataract are known to be educational and income status [5], smoking [6], diabetes [7,8,9], ultraviolet radiation [10,11,12], body mass index [13,14], estrogen replacement therapy [15,16], drug use (non-steroidal anti-inflammatory drugs) [17,18], traumatic injuries, chemicals and local diseases like uveitis or retinal detachment [19,20].

The corneal endothelium consists of a monolayer of cells on the corneal posterior surface having the main role in the transport of water from the stroma into the anterior chamber. Corneal endothelial cells are indispensable for maintaining corneal stromal hydration and tissue transparency through their ionic pump function. Unfortunately, they cannot regenerate, but they have a compensatory mechanism of repair through cellular enlargement and migration [21]. The density of endothelial cells decreases naturally with age, and a rapid cell loss occurs in the first year of life, from a cell density of approximately 6000 cells/mm^2^ during the first month of life, to 3500 cells/mm^2^ by age 5 and a range of approximately 2250–2500 cells/mm^2^ in adult life, while a minimum of 700 cells/mm^2^ are required to maintain transparency [22,23]. The remaining healthy cells gradually increase in size, which results in increased cellular pleomorphism and a decrease in the percentage of hexagonal cells with age [24]. Any traumatism and intraocular surgery cause damage in the corneal endothelium, threatening the vision. Possible damage may occur during phacoemulsification procedure, using ultrasound energy that elevates localized temperature when an excessive amount of energy is required, especially in hypermature cataracts, or if there is a longer time of phaco energy needed. The surgical technique, the anterior chamber depth and the use of ophthalmic viscoelastic devices are other factors that can influence the damage degree of the endothelium [25], as well as the type of IOLs implanted (toric versus non-toric), especially in patients with corneal degeneration or dystrophies. Studies report endothelial cell loss rates from 4% to 25% after phacoemulsification [26,27]. If cell density drops critically, corneal edema may develop, which can lead to bullous keratopathy (300–500 cells/mm^2^) [28]. Additionally, certain specific age-associated ocular diseases are considered to be correlated with recurring fall events and fracture incidence as a consequence of impaired visual acuity (cataract being the most prevalent), with significant socio-economic impact.

In this context, the aim of our study was to investigate the main outcomes of cataract surgery in terms of corneal cell density (CD), central corneal thickness (CCT) and hexagonality (HEX) of the endothelial cells in patients over 65 years of age compared to those under 65.

## 2. Materials and Methods

This study was conducted according to the principles of the Declaration of Helsinki and was approved by the Institutional Review Board of the Emergency County Hospital Oradea, Bihor County, Romania (Nr. 4238/11.02.2021). A cross-sectional, retrospective study was conducted on 120 eyes from 120 patients, out of which 77 were >65 years old (group A) and 43 were ≤65 (group B) in order to compare the corneal structure and endothelial morphological changes in terms of corneal cell density (CD), central corneal thickness (CCT) and hexagonality (HEX) of the cells after uneventful phacoemulsification cataract surgery in the Ophthalmology Department of Emergency County Hospital Oradea, Bihor County, Romania.

### 2.1. Pre-, Intra- and Postoperatively Ophthalmological Evaluation

**Figure 1:**
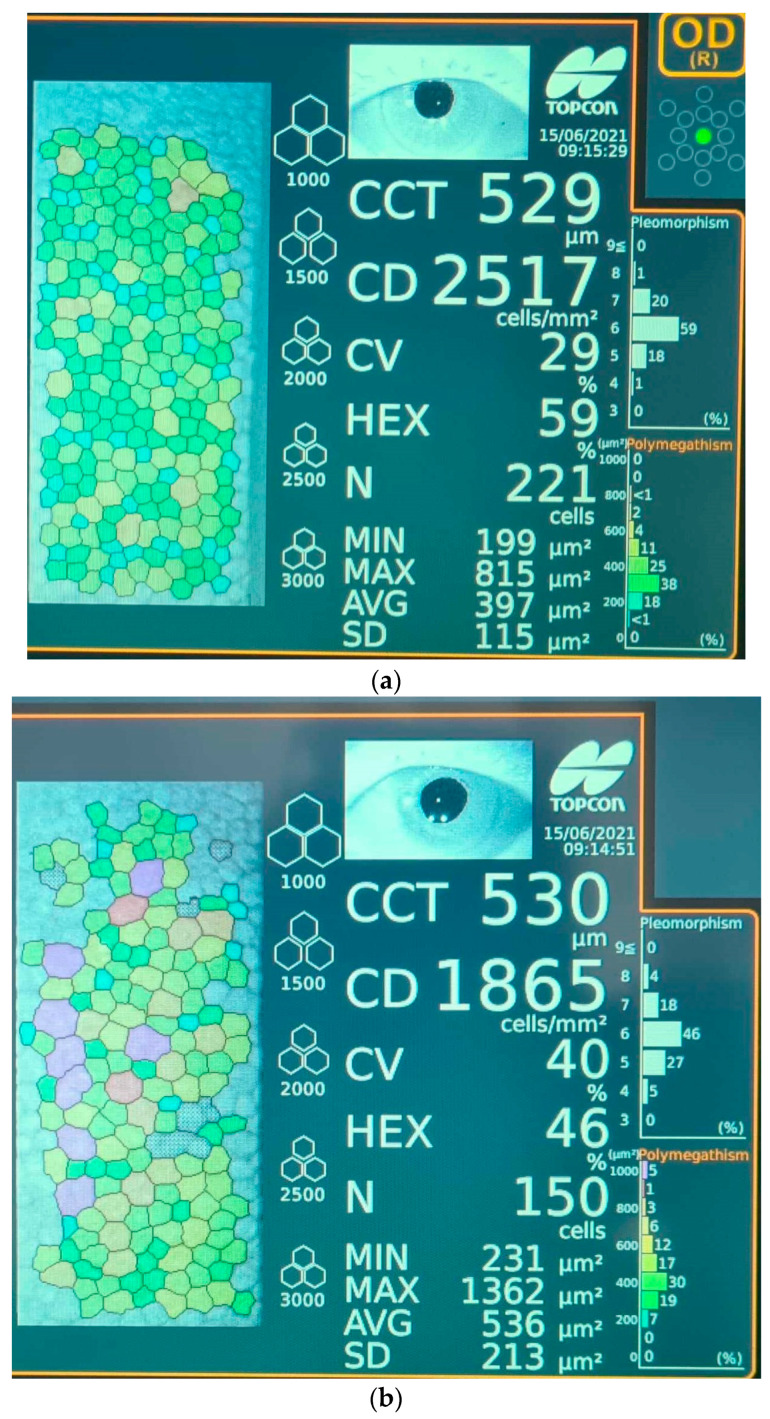
Representative images of endothelial cell layer (colored dots) 1 week after surgery performed with specular microscopy for (**a**) patient from group B (≤65 years old) and (**b**) patient from group A (>65 years old). Legend: CCT—corneal central thickness; CD—cell density; CV—coefficient of variation; HEX—cell hexagonality; N—normal value; MIN—minimum value; MAX—maximum value; AVG—average value; SD—standard deviation of specular image; Pleomorphism—percentage of cells with variation from normal hexagonal shape; Polymegathism—size variation in the endothelial monolayer.

All patients underwent a rigorous ophthalmological examination that included visual acuity, slit-lamp examination of the anterior and posterior segment, dilated fundus examination using a non-contact wide-field lens and intraocular pressure using Goldman applanation tonometry. Specular microscopy images were obtained prior to surgery, at 1 week and 4 weeks after surgery, for all study subjects. A representative photograph recorded with the specular microscope showing comparatively the main features of a cornea 1 week after surgery, in patients from groups A and B, is displayed in Figure 1. Specular microscopy was performed using a non-contact Topcon Specular Microscope (Topcon Corporation Itabashi-ku, Tokyo, Japan 2017) by recording the values of corneal endothelial cell density (CD), central corneal thickness (CCT) and percentage of hexagonality (HEX). The axial length (AXL) and anterior chamber depth (ACD) were measured before surgery with an ultrasound A-scan biometry. The ultrasound energy consumption (U/S) of the phaco machine and the effective phaco time (EPT) were observed intraoperatively (Stellaris PC, Bausch & Lomb, Rochester, NY, USA, 2016). The Stellaris phaco machine is capable of vacuum settings of up to 600 mm Hg and still maintains the chamber stability due to its ability to simultaneously monitor the flow rate and vacuum levels. Moreover, it will create feedback through the computer to control pump functions, while intraoperatively, the vacuum settings are adjusted according to individual lens hardness. 

### 2.2. Inclusion and Exclusion Criteria

**Figure 2:**
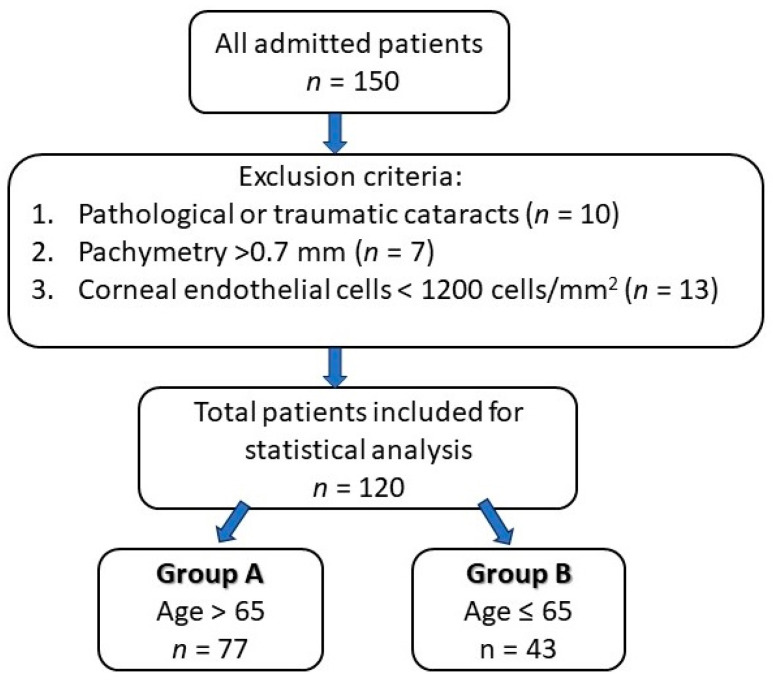
Study design chart.

The inclusion criteria of the study group consisted of patients who underwent cataract surgery by phacoemulsification technique, performed by the same surgeon (in order to minimize bias), while the exclusion criteria were patients with pathological or traumatic cataracts, pachymetry greater than 0.70 mm and an endothelial cell count less than 1200 cells/mm^2^. The study design is presented in Figure 2.

### 2.3. Surgical Procedure

The first step preoperatively was pupillary dilatation using tropicamide 1% and phenylephrine 10% followed by a peribulbar anesthesia. Two side-port incisions of 1.2 mm at the limbus and the main temporal incision of 2.2 mm were made. Viscoelastic substances were injected in the anterior chamber for space maintenance and for corneal endothelium protection. A curvilinear capsulorhexis using a coaxial capsulorhexis forceps and hydro dissection were performed by the surgeon. Emulsification of the lens using ultrasound energy and removal of the residual cortex while irrigation–aspiration was applied. The final step was IOL (non-toric monofocal intraocular lens) implantation in the capsular bag just before the excess of viscoelastic material was washed out and the corneal incisions were hydro-sealed. Application of topical ointment antibiotic–steroid combination and sterile eye patch until next day was also performed.

### 2.4. Statistical Analysis

All the statistical analyses were performed using the Stata 17.0 SE-Standard Edition (StataCorp LLC, StataCorp 4905 Lakeway Drive, College Station, Texas 77845 USA. Univariate statistical analysis was performed by applying Pearson correlation matrices and two-way ANOVA (*p* = 0.05). The factors involved in two-way ANOVA were gender with two levels, respectively F (female) and M (male), and AgeGRP with two levels, respectively ≤65 and >65. The multiple linear regression with predictions were performed for post-operative evaluation at 1 week.

## 3. Results

**Figure 3:**
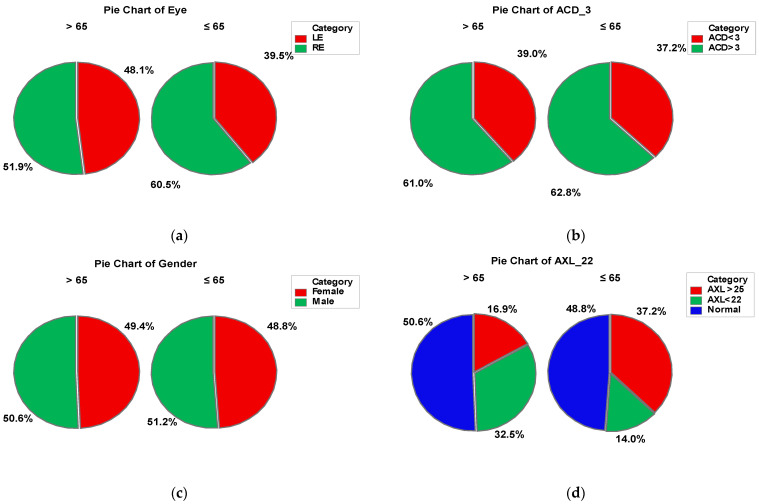
Combined socio-demographic and clinical characteristics of group A (>65 y) and B (≤65 y). Legend: LE—left eye; RE—right eye; AXL—axial length; ACD—anterior chamber depth.

This study involved 120 eyes of 120 patients undergoing cataract surgery (phacoemulsification), divided in to the following two groups: group A > 65-years old (77 patients) and group B ≤ 65-years old (43 patients). In group A, 49.4% of the patients were female and 50.6% male, while in group B, 48.8% were represented by females and 51.2% by males. The anterior chamber depth (ACD) was over 3 mm in about 60% patients from both groups. In group A, 32.5% of the patients presented values of the axial length (AXL) under 22 mm and 16.9% over 25 mm, while in group B, 37.2% of the patients had AXL values over 25 mm and 14% of them under 22 mm. In both groups, right eye was predominant (51.9% in group A and 60.5% in group B). There was no statistically significant difference between the two groups in terms of gender. The socio-demographic characteristics are presented in Figure 3.

### 3.1. Anterior Chamber Depth (ACD) and Axial Length (AXL)

The mean values for the anterior chamber depth in both groups A and B were about 3 mm, with no statistical difference between them. The mean value of the axial length in group A was 23.327 mm, while in group B was 25.040 mm, with a statistically significant difference (Table 1). When taking into consideration the factor gender–age, we noticed that females over 65 years had a mean value AXL 22.524 mm, while those under 65 years had a mean value of 26.232 mmm, but no statistical difference was noticed in males. Females over 65 years presented a mean ACD 3.064 mm, while the ones under 65 years 3.481 mm (Table 2).

### 3.2. Ultrasound Energy (US) and Effective Phaco Time (EPT)

There was a greater percentage of ultrasound energy consumption as well as a longer effective phaco time in group A, compared to group B—a mean of 11.974% of US in group A and a mean of 10.329 EPT (seconds) in group A versus their counterparts in group B of 6.927 EPT (Table 1). There was no statistically significant difference between females over and under 65 years old, nor between males over and under 65 years old in terms of ultrasound energy consumption and effective phaco time (Table 2).

### 3.3. Endothelial Cell Density (CD)

**Figure 4:**
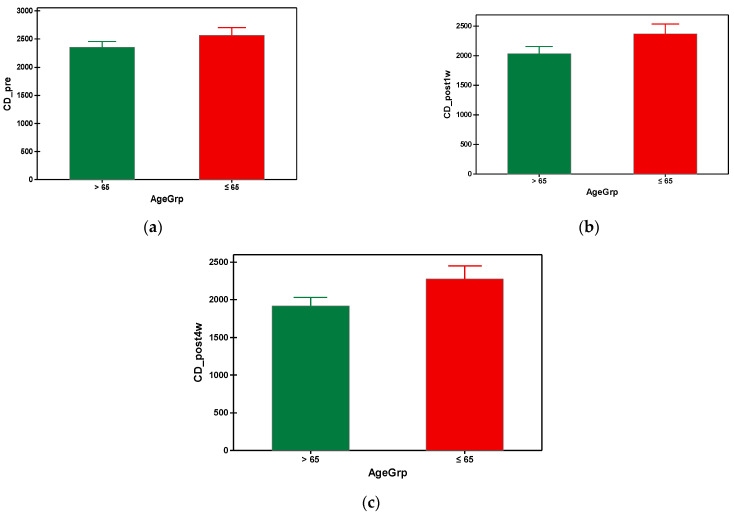
Graphical comparison means of (**a**) CD_pre, (**b**) CD_post1w and (**c**) CD_post4w parameters, with confidence intervals as error-bars, calculated for AgeGrp factor levels.

The mean preoperative cell density in group A was 2353.649 cells/mm^2^ and 2567.860 cells/mm^2^ in group B. As can be seen, the group B (with younger subjects) had a significantly higher preoperative value of cell density compared to the elderly subjects from group A (Table 3). In both groups, a progressive decrease in the endothelial cells was observed, starting from the first week post-surgical examination until the fourth postoperative week. It is also important to note that the patients from group A presented a higher loss of the endothelial cells throughout the postoperative follow-up compared to group B, which is statistically significant (Figure 4). Regarding the postoperative cell loss in group A versus group B, a significantly higher endothelial cell loss was noted in the group B after the first-week postoperative measurements (Table 4). More precisely, one week after the surgery the mean value of endothelial cell loss was 318.0519 cells/mm^2^ in group B compared to 212.6977 cells/mm^2^. Males over 65 years old had lower mean values of CD preoperatively, also postoperatively at week 1 and 4, compared to the males under 65 years old. There were no differences when taking into consideration the factor gender for females regardless of age (Table 5). The overall endothelial cell loss percentage was 13.358% in group A and 8.532% in group B.

### 3.4. Central Corneal Thickness (CCT)

**Figure 5:**
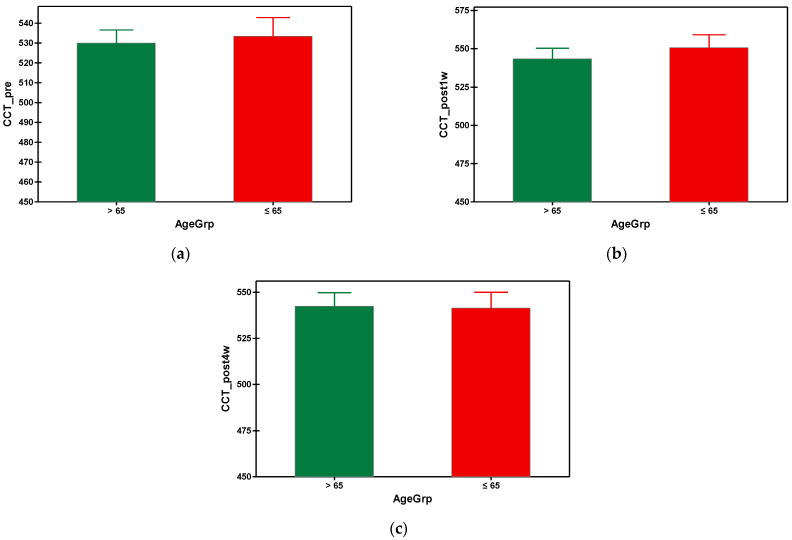
Graphical comparison means of (**a**) CCT_pre, (**b**) CCT_post1w and (**c**) CCT_post4w parameters, with confidence intervals as error-bars, calculated for AgeGrp factor levels.

After phacoemulsification, the central corneal thickness (CCT) increased in both groups, being at its maximum at the first week postoperatively, then gradually decreased until the fourth week post-surgery, when the values of CCT came back almost to the initial values (prior to surgery). In the fourth week, the mean value of CCT in group A was 542.364 µm and 541.488 µm in group B (Table 3). There was no statistical difference between the values of CCT of the group A and group B. The graphical comparisons of the means of CCT can be seen in Figure 5.

### 3.5. Hexagonal Cell Percentage (HEX)

**Figure 6:**
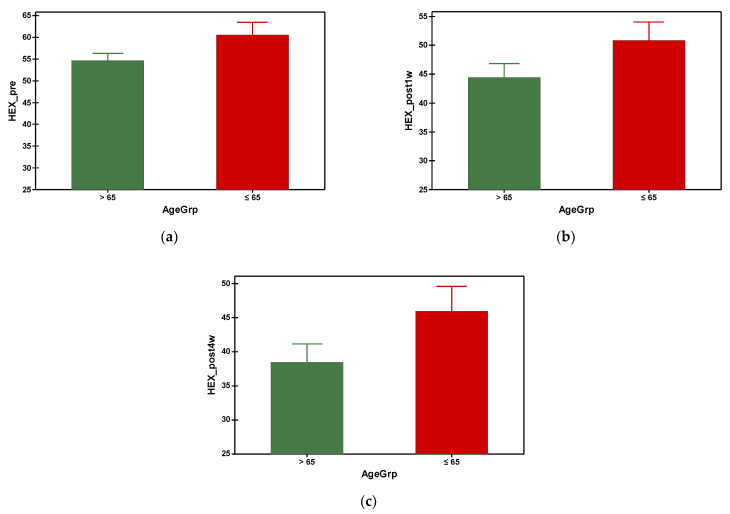
Graphical comparison means of (**a**) HEX_pre, (**b**) HEX_post1w and (**c**) HEX_post4w parameters, with confidence intervals as error-bars, calculated for AgeGrp factor levels.

The hexagonal cell percentage decreased progressively in both groups after phacoemulsification (Figure 6). The mean preoperative hexagonal cell percentage was 54.688 in group A and 60.535 in group B. It was found that group A (with older subjects) had a significantly smaller HEX at preoperative and all postoperative time points, which was statistically significant (Table 3). Referring to the gender–age factor, the hexagonal cell percentage was higher in females under 65 y compared to the ones over 65 at the preoperative measurement and at the fourth week postoperatively, but with no differences at the first week postoperatively measurement (Table 4).

### 3.6. Correlation Matrix

**Figure 7:**
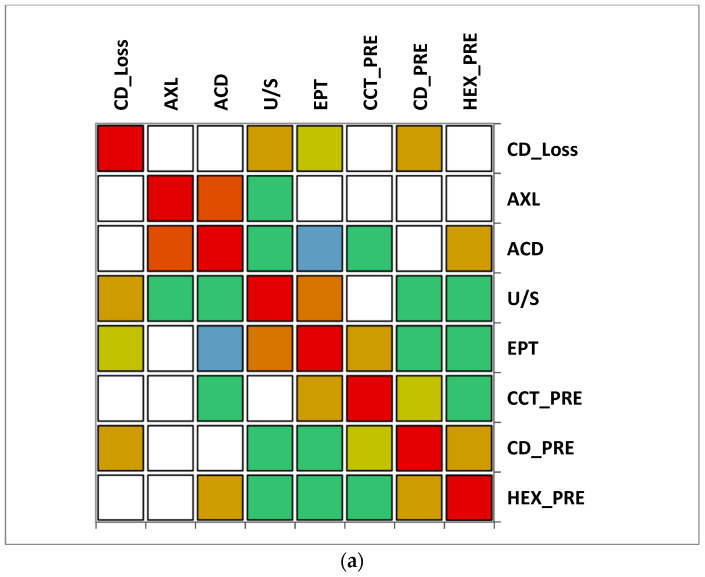

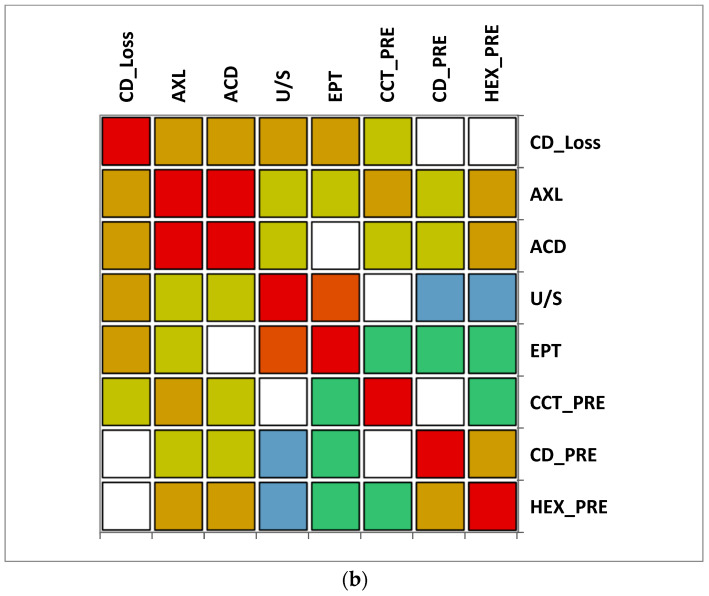
Correlation matrix between measured variables. Legend: AXL, axial length; ACD, anterior chamber depth; CCT_pre, central corneal thickness preoperative; CD_pre, cell density preoperative; HEX_pre, hexagonality of endothelial cells preoperative; CD_loss, cell density loss; U/S, ultrasound energy consumption; EPT, effective phaco time (**a**) Group A (>65); (**b**) Group B (≤65).

The correlation matrix between different variables reveals that in group A (>65 y), there was a moderate correlation between CD loss and U/S and also a moderate correlation between U/S and EPT. A moderate negative correlation was observed between ACD and EPT. In group B (≤65), a moderate positive correlation was observed between CD loss, AXL and U/S, while a very strong correlation between EPT and U/S was noticed. Additionally, a moderate negative correlation was observed between CD and U/S. In both groups, a very strong positive correlation between AXL and ACD was observed (Figure 7).

### 3.7. Multiple Regression

The prediction of the CD_post1w values by the measured parameters (AXL, ACD, U/S, EPT, CD_pre, CCT_pre, HEX_pre and Gender_f) was performed with a multiple regression model (without constant calculation option) for group A (Age > 65 y) and group B (Age ≤ 65 y) cases (Table 6). Both models are statistically significant at 0.01% level with an adjusted statistical accuracy of 96.37% and 97.50%, respectively. In group A, the U/S and CD_pre parameters have statistically significant positive coefficients, due the fact that their statistical significances are less than the 0.05 threshold. In group B, the CD_pre parameter has statistically significant positive coefficient. Despite the rest of the parameters presenting coefficients with no statistical significance at the 0.05 threshold, some of them were considered in the prediction calculations by the final multiple regression models (Table 7). This choice is a common one in classification algorithms due the fact that there can be latent independent variables interaction that can generate a higher accuracy for the experiment. In fact, the main goal in the prediction process is to gain an accuracy over 95%.

The quality of the prediction (i.e., the predicted CD_post1w values) was tested with linear regression between the CD_post1w values and the predicted ones (predicted CD_post1w). There linear regressions were analyzed without constant calculation. However, the predicted values resulted in statistically significant values (*p* < 0.0001) of 1.019773 for the Group A (Age > 65) and 0.9709181 for the Group B (Age ≤ 65), both very close to unity value; thus, the correlation between the calculated and predicted CD_post1w values are positive and strong (Table 8 and Table 9). These results also had an adjusted accuracy of 97.97% for the group A and 97.89% for the group B, both higher than the 95% proposed accuracy (Table 8 and Table 9).

**Figure 8:**
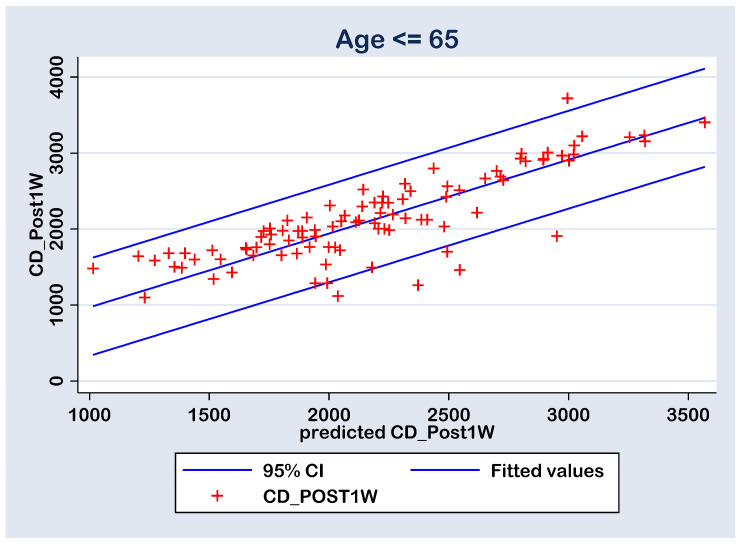
Regression line of prediction of the post-operative (CD_Post1W) cell density (multiple regression FINAL model) for the group B (≤ 65 y).

**Figure 9:**
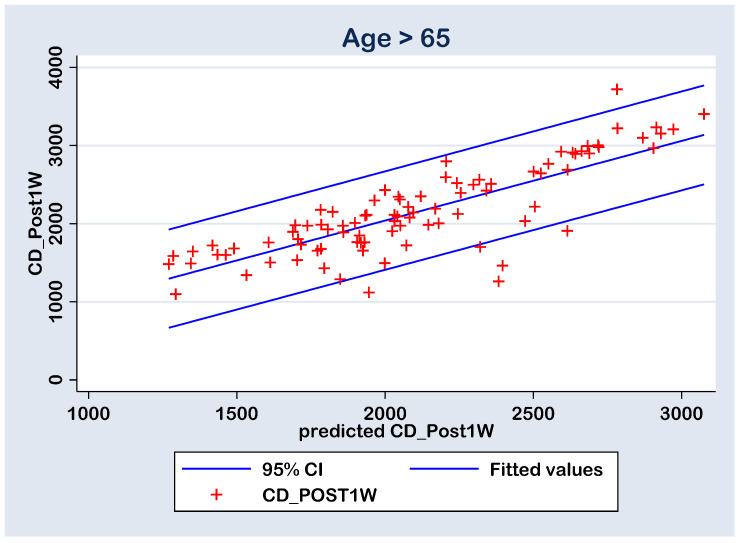
Regression line of prediction of the post-operative (CD_Post1W) cell density (multiple regression FINAL model) for the group A (>65 y).

With all this, in both cases, there are six points with high residuals (Figure 8 and Figure 9) that consist as outliers for the prediction linear regression. The reason is a rise an experimental limitation due to the small number of patients (N = 77 in group A and N = 43 in group B). Therefore, for future experiments, it is recommended to increase the sampling number above N = 120.

## 4. Discussion

In our study, we evaluated the impact of the phacoemulsification cataract surgery on the corneal endothelium in two age groups of patients, under 65 years old and over 65 years old. We decided to investigate these two categories due to the life expectancy in our country (Romania), which is 75.6 years [28]. Cataract is one of the main causes of vision loss all over the world, especially as life expectancy has increased in developed countries. Phacoemulsification cataract surgery is accepted to be the first-line treatment for cataract, with great visual recovery outcome, but we must take into consideration that the use of high-intensity ultrasound energy power for nucleus removal during surgery can lead to corneal endothelium damage [29,30,31,32]. The appearance of cataract at an early age is believed to be due to smoking, vitamin D deficiency, diabetes mellitus and high myopia. Other factors can be involved, including trauma, and endocrine, metabolic or systemic diseases [3,33,34,35]. Features of age-related cataract include ocular and/or systemic comorbidities, higher grade cataract, higher ultrasound energy consumption requirements for nucleus removal and overall, longer effective phaco time and corneal edema development. 

Our results showed a decreased in the endothelial cell count in both groups, independently of the patients age, attesting that phcoemulsification cataract surgery has traumatic effects on the corneal endothelium and this is unavoidable. Age is an independent risk factor for reduced vison after cataract surgery due to ocular tissue degeneration that combines with physiological decrease in the corneal cell density [36]. Central corneal thickness is a reliable parameter of transient corneal edema that occurs after surgical trauma. In our study, CCT increased in both groups, statistically significant in the first week post-surgery, but with values that came back to their initial measurement by week 4 post-surgery, similar with data found in other studies [37]. Hexagonality is the percentage of regular hexagonal cells to the total number of cells with different morphologies. The enlargement of the endothelial cells and the reduction of their hexagonality is a mechanism of self-repair secondary to CD loss [38]. The hexagonality of healthy cornea is about 60–70% [39]. Our results showed a dropped percentage of hexagonal cells in both groups after surgery, with a greater difference observed at week 4. Group A had a significantly smaller HEX at preoperative and all postoperative time points, compared to group B.

Many studies claim that ACD influences the CD loss, while others states that ACD has no influence on the corneal endothelium damage [26,40]. Hypothetically, if ACD is shallow, damage of the corneal endothelium may occur during cataract surgery, as all surgical maneuvers take place closer to the endothelium. Walkow et al. [40] showed that a shallow ACD associates with a higher rate of endothelial cell loss. Also, McCarey et al. [41] concluded that endothelial cells were affected in different degrees by the manipulation of intraoperative surgical instruments in eyes with shallow ACDs. Hyung Bin Hwang et al. [42] emphasized in their study that eyes with shallow ACDs, especially when associated with hard cataract, are prone to more corneal endothelial cell loss, and they found a mean endothelial cell count of 12.94 ± 3.16%. On the other hand, neither O’Brien et al. [26], nor Reuschel et al. [43] found a relationship between ACD or AXL and the endothelial cell loss. In our study, the mean value of ACD in both of our groups was about 3 mm, and we did not identify ACD as a risk factor of postoperative endothelial cell loss. Regarding the length of the eye, we report a mean value of 25.040 mm in group B versus 23.327 mm in group A. An axial length > 25 mm correlates with myopia (high myopia > 26 mm AXL). One of the complications of myopia is the early onset of cataract (presenile cataract) [44], and this may be an explication for the predominance of a higher axial length among the patients under 65 years (group B) and hypothetically possessing a larger room for surgical instruments manipulation during phacoemulsification could be responsible for a lower endothelial cell loss.

It is known that phacoemulsification cataract surgery comes with a 5–20% loss of corneal endothelial cells [23]. The percentage of endothelial cell loss in our study falls within the limits of the existing studies, 13% in group A and 8% in group B. Higher cell loss after phaco was found to be correlated with older age [45], confirmed also by the results of our study. Gogate et al. demonstrated higher rates of endothelial cell loss in mature cataracts and white cataracts, with values of about 18% [46]. In this context, greater attention should be paid to elderly patients, who, by default, present lower values of cell density and changes in the corneal endothelium as well as harder nucleus, which requires higher phaco values and longer phaco time to remove the cataractogenous lens. Specular microscopy is indispensable in the case of corneal degenerations or dystrophies or suspicions of them. Additionally, cataract surgery in elderly patients should be also related to their functional vision assessment, daily needs and individual priorities [47].

The IOLs implanted in the current study were non-toric monofocals. However, comparing the different types of IOLs, some authors found that there is a significant loss of ECD in eyes implanted with a toric IOL during live surgery [48]. They also highlighted the importance and the benefits of live view in terms of medical education and the low risk of complications. Yamauchi et al. found that toric IOLs are able to reduce spectacle dependency more than non-toric ones [49]. A further study will be dedicated to investigate the differences between the outcomes of cataract surgery using toric versus non-toric IOL implantation.

This study has few limitations. First, it was conducted on a small sample. Larger sample size can provide much more credibility to the results. The second limitation of our study was the retrospective nature based on data from a single hospital. Relatively short follow-up duration might be another limitation, while extended follow-up might have clarified more post-surgical complications including posterior capsule opacity and lens dislocation.

## 5. Conclusions

Our study evaluated the influence of phacoemulsification cataract surgery technique on corneal cell density, central corneal thickness and hexagonality preoperatively and postoperatively at 1 and 4 weeks using the specular microscope. The effect of gender, axial length and anterior chamber depth on the parameters of corneal endothelium in the two groups, under and over 65 years of age were evaluated. There were significant differences between the preoperative and postoperative cell density values in both groups, with a progressive loss of cells until the fourth week of evaluation. A significant increase in the CCT was observed in both groups after the first week, while the initial values were restored to almost normal by the fourth week post-surgery, with no statistical difference between group A and B. The cell hexagonality suffered a progressive drop in both groups, with significantly lower HEX values at all preoperative and postoperative times in group A (>65 y). Although the cell density loss was significant in both groups, but more advanced in older patients, accompanied with increased central corneal thickness and reduced values of hexagonality, we had no cases of endothelial decompensation secondary to surgical trauma, characterized by epithelial and subepithelial bullae and stromal edema (also known as bullous keratopathy) that is directly responsible for vision loss following cataract surgery. This outcome can be associated to a good preoperative assessment of the patient, together with the surgeon’s experience and the application of viscoelastic substances with protective role on the corneal endothelium.

Phacoemulsification can and truly does improve the visual function of all patients, under and over 65 years old with senile or presenile cataracts, giving them fully independence for daily activities, improving their life quality. The use of specular microscopy in all patients undergoing phacoemulsification and intraoperative proper use of viscoelastic substances for endothelium protection, as well as making adjustments of intraoperative parameters used for nucleus removal according to preoperative cell density values and caution in terms of phaco power intensity and ultrasound energy, for a successful cataract surgery are all strongly recommended.

## Figures and Tables

**Table 1:** Descriptive statistics of AXL, ACD, US and EPT parameters, as means with standard deviations calculated for AgeGrp factor levels. Means were compared within two-way ANOVA (*p* = 0.05), in a post hoc pairwise comparison Dunn–Sidak test (*p* = 0.05). Different letters denote statistically significant different means.

No. Patients	AgeGrp	AXL	ACD	U/S	EPT
**77**	**>65**	23.327 b ± 2.588	3.146 a ± 0.582	11.974 a ± 4.193	10.329 a ± 5.670
**43**	**≤65**	25.040 a ± 3.409	3.314 a ± 0.518	9.674 b ± 4.648	6.927 b ± 5.839

Legend: AgeGrp (age group); AXL—axial length; ACD—anterior chamber depth; U/S—ultrasound energy consumption; EPT—effective phaco time.

**Table 2:** Descriptive statistics of AXL, ACD, US and EPT parameters, as means with standard deviations calculated for Gender_AgeGrp factor levels. Means were compared within two-way ANOVA (*p* = 0.05), in a post hoc pairwise comparison Dunn–Sidak test (*p* = 0.05). Different letters denote statistically significant different means.

No. Patients	Gender_AgeGrp	AXL	ACD	U/S	EPT
38	Female_ > 65	22.524 ^b^ ± 2.174	3.064 ^b^ ± 0.573	12.211 ^a^ ± 4.140	10.258 ^a^ ± 5.678
21	Female_ ≤ 65	26.232 ^a^ ± 3.525	3.481 ^a^ ± 0.468	10.619 ^a,b^ ± 5.296	7.610 ^a^ ± 7.127
39	Male_ > 65	24.111 ^b^ ± 2.742	3.225 ^a,b^ ± 0.587	11.744 ^a,b^ ± 4.284	10.397 ^a^ ± 5.735
22	Male_ ≤ 65	23.902 ^b^ ± 2.938	3.154 ^a,b^ ± 0.523	8.773 ^b^ ± 3.841	6.275 ^a^ ± 4.348

Legend: Gender_AgeGrp (gender age group); AXL—axial length; ACD—anterior chamber depth; U/S—ultrasound energy consumption; EPT—effective phaco time. Different letters denote statistically significant different means.

**Table 3:** Descriptive statistics of CD_pre, CD_post1w, CD_post4w, CCT_pre, CCT_post1w, CCT_post4w, HEX_pre, HEX_post1w and HEX_post4w parameters, as means with standard deviations calculated for AgeGrp factor levels. Means were compared within two-way ANOVA (*p* = 0.05), in a post hoc pairwise comparison Dunn–Sidak test (*p* = 0.05)–different letters denote statistically significant different means.

No. Patients	AgeGrp	CD_pre	CD_post1w	CD_post4w
77	>65	2353.649 ^b^ ± 458.247	2036.169 ^b^ ± 517.853	1916.143 ^b^ ± 501.560
43	≤65	2567.860 ^a^ ± 426.972	2372.581 ^a^ ± 544.728	2274.581 ^a^ ± 572.560
No. patients	AgeGrp	CCT_pre	CCT_post1w	CCT_post4w
77	>65	530.156 ^a^ ± 28.807	543.494 ^a^ ± 29.625	542.364 ^a^ ± 32.693
43	≤65	533.558 ^a^ ± 30.726	550.651 ^a^ ± 27.498	541.488 ^a^ ± 27.812
No. patients	AgeGrp	HEX_pre	HEX_post1w	HEX_post4w
77	>65	54.688 ^b^ ± 7.212	44.416 ^b^ ± 10.710	38.442 ^b^ ± 12.052
43	≤65	60.535 ^a^ ± 9.349	50.860 ^a^ ± 10.426	45.977 ^a^ ± 11.903

Legend: AgeGrp—age group; CD_PRE—cell density preoperative; CD_post1w—cell density 1 week postoperative; CD_post4w-cell density 4 weeks postoperative; CCT_pre—central corneal thickness preoperative; CCT_post1w—central corneal thickness 1 week postoperative; CCT_post4w—central corneal thickness 4 weeks postoperative; HEX_pre—hexagonality preoperative; HEX_post1w—hexagonality 1 week postoperative; HEX_post4w—hexagonality 4 weeks postoperative. Different letters denote statistically significant different means.

**Table 4:** Endothelial cell loss (cell/mm^2^) (mean ± SD).

	Group A (>65)	Group B (≤65)	*p* Value (Group A > 65 vs. Group B ≤ 65)
**CD_pre**	2353.649 ^b^ ± 458.247	2567.860 ^a^ ± 426.972	0.013
**CD_post1w**	2036.169 ^b^ ± 517.853	2372.581 ^a^ ± 544.728	0.001
**Endothelial cell loss (n)**	318.0519 ^a^ ± 212.6977	212.6977 ^a^ ± 261.7497	0.1460
**Endothelial Cell loss %**	13.358 ^a^ ± 8.5320	8.532 ^b^ ± 9.5452	0.0428

Legend: CD_pre—cell density preoperative; CD_post1w—cell density 1 week postoperative.

**Table 5:** Descriptive statistics of CD_pre, CD_post1w, CD_post4w, CCT_pre, CCT_post1w, CCT_post4w, HEX_pre, HEX_post1w and HEX_post4w parameters, as means with standard deviations calculated for Gender_AgeGrp factor levels. Means were compared within **two-way** ANOVA (*p* = 0.05), in a post hoc pairwise comparison Dunn–Sidak test (*p* = 0.05)–different letters denote statistically significant different means.

No. patients	Gender_AgeGrp	CD_pre	CD_post1w	CD_post4w
38	Female_ > 65	2435.553 ^a,b^ ± 524.287	2091.026 ^b^ ± 621.578	1955.763 ^b^ ± 581.763
21	Female_ ≤ 65	2507.714 ^a,b^ ± 441.443	2256.571 ^a,b^ ± 622.338	2151.571 ^a,b^ ± 627.473
39	Male_ > 65	2273.846 ^b^ ± 372.953	1982.718 ^b^ ± 392.695	1877.538 ^b^ ± 412.896
22	Male_ ≤ 65	2625.273 ^a^ ± 414.665	2483.318 ^a^ ± 445.299	2392.000 ^a^ ± 501.099
No. patients	Gender_AgeGrp	CCT_pre	CCT_post1w	CCT_post4w
38	Female_ > 65	533.132 ^a^ ± 30.521	547.184 ^a^ ± 33.706	544.105 ^a^ ± 37.429
21	Female_ ≤ 65	535.429 ^a^ ± 33.345	549.571 ^a^ ± 25.443	540.381 ^a^ ± 26.352
39	Male_ > 65	527.256 ^a^ ± 27.114	539.897 ^a^ ± 24.943	540.667 ^a^ ± 27.706
22	Male_ ≤ 65	531.773 ^a^ ± 28.679	551.682 ^a^ ± 29.891	542.545 ^a^ ± 29.719
No. patients	Gender_AgeGrp	HEX_pre	HEX_post1w	HEX_post4w
38	Female_ > 65	54.632 ^b^ ± 7.492	45.395 ^a,b^ ± 11.081	39.184 ^b^ ± 12.781
21	Female_ ≤ 65	61.524 ^a^ ± 9.169	52.905 ^a^ ± 9.823	49.714 ^a^ ± 10.311
39	Male_ > 65	54.744 ^b^ ± 7.025	43.462 ^b^ ± 10.389	37.718 ^b^ ± 11.418
22	Male_ ≤ 65	59.591 ^a,b^ ± 9.635	48.909 ^a,b^ ± 10.832	42.409 ^a,b^ ± 12.443

Legend: Gender_AgeGrp—gender age group; CD_pre—cell density preoperative; CD_post1w—cell density 1 week postoperative; CD_post4w-cell density 4 weeks postoperative; CCT_pre—central corneal thickness preoperative; CCT_post1w—central corneal thickness 1 week postoperative; CCT_post4w—central corneal thickness 4 weeks postoperative; HEX_pre—hexagonality preoperative; HEX_post1w—hexagonality 1 week postoperative; HEX_post4w—hexagonality 4 weeks postoperative. Different letters denote statistically significant different means.

**Table 6:** Univariate association of the measured variables with the cell density loss.

	Group A (>65 y)		Group B (≤65 y)	
	Correlation Coefficient (R)	*p* Value	Correlation Coefficient (R)	*p* Value
**AXL**	0.2038	0.2197	0.3405	0.1309
**ACD**	0.1289	0.4406	0.2078	0.3660
**U/S**	0.2720	0.0985	0.2987	0.1885
**EPT**	0.2265	0.1714	0.3518	0.1179
**CCT_pre**	0.2325	0.1600	0.1872	0.4165
**CD_pre**	0.1384	0.4073	−0.0424	0.8552
**HEX_pre**	0.0589	0.7253	−0.1466	0.5260

Legend: AXL—axial length; ACD—anterior chamber depth; CCT_pre—central corneal thickness preoperative; CD_pre—cell density preoperative; HEX_pre—hexagonality preoperative; U/S—ultrasound energy consumption; EPT—effective phase time.

**Table 7:** Multiple regression FINAL model of the selected variables with the post-operative (CD_Post1W) cell density.

	Group A (>65 y)				Group B (≤65 y)		
Factor	Coefficient	Standard error	*p* Value	Factor	Coefficient	Standard Error	*p* Value
U/S	−24.2216	9.136519	0.0100 *	AXL	−24.9617	12.48921	0.0530 *
CD_pre	0.827274	0.083201	0.0000 *	U/S	−12.3783	9.477861	0.1990
CCT_pre	0.71207	0.45167	0.1190 *	CD_pre	1.059313	0.099469	0.0000 *
				HEX_pre	6.503726	4.942817	0.1960

Legend: AXL, axial length; ACD, Anterior chamber depth; U/S, ultrasound energy consumption; EPT, effective phaco time. * *p* < 0.05.

**Table 8:** Prediction of the post-operative (CD_Post1W) cell density with the multiple regression FINAL model, AGE ≤ 65 case.

**Prob > F = 0.0000**				
**R-squared = 0.9791**				
**Adj R-squared = 0.9789**				
**CD_post1w**	**Coefficient**	**Std. err.**	**t**	***p* > |t|**
**pred_CD_1w, Age ≤ 65**	0.9709181	0.013	74.69	0.0000

**Table 9:** Prediction of the post-operative (CD_Post1W) cell density with the multiple regression FINAL model, AGE > 65 case.

**Prob > F = 0.0000**				
**R-squared = 0.9798**				
**Adj R-squared = 0.9797**				
**CD_post1w**	**Coefficient**	**Std. err.**	**t**	***p* > |t|**
**pred_CD_1w_Age > 65**	1.019773	0.013408	76.06	0.0000

## Data Availability

The data supporting the reported results are available in the medical archive of Emergency County Hospital Oradea, Bihor, Romania.
